# Characteristics of common pathogens of urogenital tract among outpatients in Shanghai, China from 2016 to 2021

**DOI:** 10.3389/fpubh.2023.1228048

**Published:** 2023-11-27

**Authors:** Su Wang, Li Ding, Yixin Liu, Zhaoyang Sun, Wenrong Jiang, Yingxin Miao, Shiwen Wang, Jun Meng, Hu Zhao

**Affiliations:** ^1^Department of Laboratory Medicine, Huadong Hospital Affiliated to Fudan University, Shanghai, China; ^2^Shanghai Key Laboratory of Clinical Geriatric Medicine, Huadong Hospital Affiliated to Fudan University, Shanghai, China; ^3^Research Centre on Aging and Medicine, Fudan University, Shanghai, China; ^4^Department of Laboratory Medicine, The First Affiliated Hospital, Zhejiang University School of Medicine, Hangzhou, China; ^5^Department of Laboratory Medicine, Ruijin Hospital, Shanghai Jiao Tong University School of Medicine, Shanghai, China

**Keywords:** *Ureaplasma urealyticum*, *Chlamydia trachomatis*, *Neisseria gonorrhoeae*, urogenital tract, outpatients

## Abstract

**Background:**

*Ureaplasma urealyticum, Chlamydia trachomatis*, and *Neisseria gonorrhoeae* are the prevalent causes of several genital diseases worldwide; however, their characteristics in different genders have not been well documented in Shanghai. The aim of this study is to describe the prevalence of common pathogens among outpatients, considering variations by gender and age.

**Methods:**

From January 1, 2016, to December 31, 2021, the urogenital swabs of 16216 outpatients aged 3–95 years from two general hospitals in Shanghai were collected. All participants' swabs were investigated for *U. urealyticum, C. trachomatis*, and *N. gonorrhoeae* by isothermal RNA-based simultaneous amplification and testing. The basic information of all participants was also recorded, including age and gender. The chi-square test was used to compare the prevalence between different genders, age groups, and infection patterns.

**Results:**

There were 5,744 patients (35.42%) with positive samples whose ages ranged from 7 to 80 years (33.23 ± 8.63 years), and 62.14% of them were women. The most common pathogen detected was *U. urealyticum* (85.08%). The highest prevalence rate of all three pathogens was found in patients aged ≤ 20 years (40.53%, 95% confidence intervals [CI]: 33.80%-47.63%). The prevalent rate of *U. urealyticum* was higher in men (33.36%, 95% CI: 32.19%-34.55%). The overall prevalence rates of *U. urealyticum, C. trachomatis*, and *N. gonorrhoeae* were 30.14% (95% CI: 29.44%-30.85%), 6.00% (95% CI: 5.64%-6.38%), and 2.10% (95% CI: 1.89%-2.33%).

**Conclusions:**

*Ureaplasma urealyticum* was the most prevalent pathogen in the population, and its prevalence decreased with age. Young men aged ≤ 20 years were more frequently infected. Regular screening for sexually transmitted pathogens in different genders and age groups are warranted, particularly in young men.

## 1 Introduction

Sexually transmitted infection (STI) is one of the most common communicable conditions and has a profound impact on people's sexual and reproductive health (1, 2). More than 1 million new STI cases are reported each day worldwide, of which 50% occurred among young patients (3). *Ureaplasma urealyticum* (UU), *Chlamydia trachomatis* (CT), and *Neisseria gonorrhoeae* (NG) are the most common causative pathogens leading to bacterial STIs, and have gained increasing notoriety owing to their high prevalence (3, 4). About 70% of the sexually active individuals presented with UU in an ongoing carrier state (5). UU is a common commensal colonizing the genitourinary tract and often infects young people after unsafe sexual intercourse. UU infection can lead to infertility, abortion, and other fertility problems in women of childbearing age (6, 7). CT is an intracellular pathogen with an infection rate of 9.8% (8, 9) and of various serotypes (10). NG is the only one among the three pathogens that can be observed under the microscope by gram staining (11) and usually causes urethritis and cervicitis (9).

Timely and effective epidemiologic surveillance can help to make policies for the prevention, screening, and treatment of STIs (3, 12). In China, only a few studies have been conducted to monitor the prevalence of STI pathogens. For instance, a research conducted in Sichuan, which exclusively included female patients seeking care at the Department of Obstetrics and Gynecology, found that UU was identified as the leading cause of STIs (13). However, men are also important reservoirs of STI pathogens, and men who have sex with men (MSM) are seriously affected by STIs because of their particular sexual behavior (3). Therefore, a better understanding of the infection status and epidemiologic characteristics of these pathogens in both men and women is necessary.

This study included 16,216 outpatients attending healthcare facilities from January 1, 2016 to December 31, 2021 to update the prevalence and characteristics of UU, CT, and NG infections in Shanghai. We investigated the distribution of these species in different ages and genders. To our best knowledge, there is no study from Shanghai describing the epidemiologic characteristics of STI-related pathogens separately in men and women over such a long period.

## 2 Materials and methods

### 2.1 Settings

The study was conducted in two hospitals, namely Ruijin Hospital and Huadong Hospital. Ruijin Hospital, Shanghai Jiao Tong University School of Medicine is a tertiary university-affiliated general hospital, located in the Huangpu district of Shanghai, which is a large metropolitan region with over 25 million inhabitants in China. It is a general 2200-bed hospital with emergency department, intensive care unit (ICU), surgery department and other departments, providing services to ~2.7 million patients annually. Huadong Hospital affiliated to Fudan University is also a tertiary university-affiliated general hospital in Jing'an district with 1,400 beds and different medical departments.

### 2.2 Study design

This descriptive and cross-sectional study was performed from January 2016 to December 2021 in these hospitals located in two different districts with the largest flow of people in the central city of Shanghai.

### 2.3 Participants recruitment

The participants were outpatients who came to Ruijin Hospital and Huadong Hospital from January 2016 to December 2021 to measure the presence of UU, CT, and NG infections. In total, 16,216 participants were enrolled according to the following inclusion criteria: (1) being outpatients during the study period; and (2) willing to provide urogenital tract samples. Pregnant women, patients treated for STIs within 2 weeks before testing, and those with a contraindication were excluded. All participants completed a data collection form to specify their basic information, including age, gender, clinical department, and diagnosis. The collected data were entered into a dataset in the laboratory information system (LIS) of the Department of Laboratory Medicine. Only the first positive test was reviewed and recorded for each outpatient per year.

### 2.4 Data collection and measurement

The urogenital swabs of all participants were sampled by physicians. The samples were promptly put into the cell preservation medium to prevent the degradation of RNA samples. It was stored at 2–8°C and processed within 24 h using commercial kits (Shanghai Rendu Biotechnology Co., Ltd). The simultaneous amplification and testing (SAT) method was employed to detect UU, CT, and NG in each sample (14). The SAT method was divided into RNA extraction and amplification and was conducted commercial kits of Rendu Biotechnology Co., Ltd (Shanghai, China) (15). The RNA ion was extracted by the magnetic bead method. Then, a 30 μL RNA sample was mixed with 10 μL enzyme reagents as the 40 μL final system for amplification. All reactions were performed on the ABI 7500 detection system (Life Technologies, CA, USA) in the following conditions: 42°C for 60 s, 40 cycles. The target genes and internal controls were respectively detected by FAM and VIC fluorescent dyes. When a clear melting curve of the expected site (dt ≤ 35, Tm = 68 ± 5°C) was obtained, the results were considered positive. The lowest detection limit of the kit was 10^3^ copies/test.

### 2.5 Data management and analysis

Two authors (SW and JM) independently extracted participants' data from LIS to ensure authenticity. We extracted data on gender, age, and detection results and recorded the number of participants with missing values. If the information was incomplete, we extracted the methods of imputation and any reported reasons for missing data. Disagreement was resolved by discussion with a third author. Descriptive statistics were used to describe the study population, frequency data, and the associations between variables (genders and age groups). The age groups were divided into ≤ 20, 21–30, 31–40, 41–50, 51–60, and >60 years. SAS 9.4 (SAS Institute Inc., Cary, NC, USA) was employed for statistical analysis. The percentages were calculated for categorical data. A 95% confidence interval (CI) was used to estimate prevalence rates, and the mean and standard deviation (mean ± SD) were used for continuous variables. Categorical variables were analyzed using Pearson's chi-square test with Fisher's exact test as appropriate. The prevalence trends in age groups were compared using Cochran-Armitage Test. A two-tailed *p* < 0.05 was considered statistically significant.

## 3 Results

### 3.1 Characteristics of participants

The ages of enrolled 16,216 participants ranged from 3 to 95 years (34.10 ± 9.29 years), and most participants were women (10,076, 62.14%). Most of the tested patients were 21–40 years old (13,288, 81.94%), and the highest number of tests was found in 2021 (5,447, 33.59%). Except for the Department of Gynecology and Andrology, the majority of patients were from the Department of Urology (3,985, 24.57%) and Reproductive Center (3,715, 22.91%) (Supplementary Table S1).

In total, 5,744 patients were infected (5,744, 35.42%), whose ages ranged from 7 to 80 years (33.23 ± 8.63 years). Patients were predominantly aged 21–30 (2,435, 42.39%) and 31–40 (2,290, 39.87%) years, and the highest positive detection rate (PDR) was in the ≤ 20 group (53.68%). Women constituted a greater proportion of patients (3,170, 55.19%), but the PDR among women was markedly lower than that in men (31.46 vs. 41.92%). Infertility was the most common complication in both men (25.33%) and women (17.57%) (Table 1).

**Table 1:** The general characteristics of 5,744 positive patients.

**Group**	**Total**			**Male**			**Female**			***p***
	***N***	**Proportion (%)**	**PDR (%)**	***N***	**Proportion (%)**	**PDR (%)**	***N***	**Proportion (%)**	**PDR (%)**	
**Total**	**5,744**	**100.00%**	**35.42%**	**2,574**	**44.81%**	**41.92%**	**3,170**	**55.19%**	**31.46%**	**<0.0001**
**Infection types**										
Single	5316	92.55%	32.78%	2,330	43.83%	37.95%	2,986	56.17%	29.63%	<0.0001
Double	400	6.96%	2.47%	224	56.00%	3.65%	176	44.00%	1.75%	<0.0001
Triple	28	0.49%	0.17%	20	71.43%	0.33%	8	28.57%	0.08%	0.0045
**Age Groups**										
≤ 20	102	1.78%	53.68%	44	43.14%	52.38%	58	56.86%	54.72%	0.7315
21–30	2,435	42.39%	38.76%	1,107	45.46%	44.55%	1,328	54.54%	34.97%	0.3954
31–40	2,290	39.87%	32.69%	1,099	47.99%	42.53%	1,191	52.01%	26.94%	<0.0001
41–50	621	10.81%	36.34%	237	38.16%	39.17%	384	61.84%	34.78%	0.0004
51–60	218	3.80%	35.28%	55	25.23%	28.80%	163	74.77%	38.17%	<0.0001
>60	78	1.36%	18.98%	32	41.03%	16.75%	46	58.97%	20.91%	0.4984
**Department**										
Gynecology	2,337	40.69%	31.71%	-	-	-	2,337	100%	31.71%	-
Urology	1,749	30.45%	43.89%	1,552	88.74%	44.12%	197	11.26%	42.18%	<0.0001
Reproductive Center	1,235	21.50%	33.24%	658	53.28%	40.10%	577	46.72%	27.82%	<0.0001
Andrology	264	4.60%	38.60%	264	100.00%	38.60%	-	-	-	-
Physical Examination Center	100	1.74%	31.25%	93	93.00%	34.19%	7	7.00%	14.58%	<0.0001
Pain	47	0.82%	47.47%	1	2.13%	100.00%	46	97.87%	46.94%	<0.0001
Others	12	0.21%	27.91%	6	50.00%	25.00%	6	50.00%	31.58%	0.7175
**Clinical symptoms or complications**										
Urinary tract infection	1,746	30.40%	46.03%	1,389	80%	46.44%	357	20%	44.51%	<0.0001
Gynecological inflammation	1,343	23.38%	56.15%	-	-	-	1,343	100%	56.15%	-
Infertility	1,209	21.05%	19.64%	652	54%	39.76%	557	46%	12.33%	<0.0001
Physical examination	757	13.18%	29.28%	146	19%	16.67%	611	81%	35.75%	<0.0001
Diseases of prostate	312	5.43%	75.73%	312	100%	75.73%	-	-	-	-
Irregular menstruation	133	2.32%	48.36%	-	-	-	133	100%	48.36%	-
Gynecological tumors	74	1.29%	43.79%	-	-	-	74	100%	43.79%	-
Abdominal pain	53	0.92%	49.53%	0	0%	0%	53	100%	50.00%	<0.0001
Diseases of foreskin and penis	48	0.84%	31.79%	48	100%	31.79%	-	-	-	-
Adverse pregnancy	19	0.33%	38.00%	-	-	-	19	100%	39.58%	-
Urinary tract tumor	14	0.24%	37.84%	0	0%	0%	14	100%	51.85%	0.0007
Urolithiasis	13	0.23%	39.39%	12	92%	40.00%	1	8%	33.33%	0.0006
Sexual dysfunction	8	0.14%	50.00%	8	100%	50.00%	-	-	-	-
Others	15	0.26%	38.46%	7	46.67%	63.64%	8	53.33%	28.57%	0.8850

### 3.2 Prevalence of UU, CT, and NG

Among the infected cases, 4,887 were positive for UU (85.08%), 973 for CT (16.94%), and 340 for NG (5.92%). Patients with UU infection were predominantly aged 21–30 years (41.99%) and were mostly women (58.09%). Among all participants, the overall prevalence of UU was 30.14% (95% CI: 29.44–30.85%), which was significantly higher than that of CT (6.00%, 95% CI: 5.64%-6.38%) or NG (2.10%, 95% CI: 1.89%-2.33%) (*p* < 0.0001). In men, the PDRs of UU, CT, and NG were 33.36% (95% CI: 32.19–34.55%), 8.36% (7.69–9.08%), and 4.51% (4.02–5.06%), respectively. Among women, the PDRs of UU, CT, and NG were 28.18% (27.31–29.07%), 4.57% (4.18–5.00%), and 0.63% (0.49–0.80%), respectively (Table 2).

**Table 2:** The prevalence and distribution of genders in UU, CT, and NG positive patients in different genders and age groups.

**Age groups**	**Total**				**Male**				**Female**				***p***
	***N***	**Proportion (%)**	**PDR [95% CI] (%)**		***N***	**Proportion (%)**	**PDR [95% CI] (%)**		***N***	**Proportion (%)**	**PDR [95% CI] (%)**		
**UU**	**4,887**	**100%**	**30.14%**	**[29.44–30.85%]**	**2,048**	**41.91%**	**33.36%**	**[32.19–34.55%]**	**2,839**	**58.09%**	**28.18%**	**[27.31–29.07%]**	**<0.0001**
≤ 20	77	1.58%	40.53%	[33.80–47.63%]	27	35.06%	32.14%	[23.12–42.72%]	50	64.94%	47.17%	[37.93–56.60%]	0.2200
21–30	2,052	41.99%	32.66%	[31.51–33.83%]	879	42.84%	35.37%	[33.51–37.27%]	1,173	57.16%	30.88%	[29.43–32.37%]	0.2627
31–40	1,952	39.94%	27.87%	[26.83–28.93%]	894	45.80%	34.60%	[32.79–36.46%]	1,058	54.20%	23.93%	[22.7–25.21%]	<0.0001
41–50	544	11.13%	31.83%	[29.66–34.08%]	181	33.27%	29.92%	[26.41–33.69%]	363	66.73%	32.88%	[30.17–35.71%]	<0.0001
51–60	191	3.91%	30.91%	[27.39–34.66%]	40	20.94%	20.94%	[15.77–27.25%]	151	79.06%	35.36%	[30.97–40.01%]	<0.0001
>60	71	1.45%	17.27%	[13.92–21.22%]	27	38.03%	14.14%	[9.90–19.79%]	44	61.97%	20.00%	[15.25–25.78%]	0.5046
**CT**	**973**	**100%**	**6.00%**	**[5.64–6.38%]**	**513**	**52.72%**	**8.36%**	**[7.69–9.08%]**	**460**	**47.28%**	**4.57%**	**[4.18–5.00%]**	**<0.0001**
≤ 20	36	3.70%	18.95%	[14.01–25.12%]	17	47.22%	20.24%	[13.04–30.04%]	19	52.78%	17.92%	[11.78–26.30%]	0.5005
21–30	470	48.30%	7.48%	[6.86–8.16%]	238	50.64%	9.58%	[8.48–10.80%]	232	49.36%	6.11%	[5.39–6.92%]	0.2079
31–40	356	36.59%	5.08%	[4.59–5.62%]	191	53.65%	7.39%	[6.44–8.46%]	165	46.35%	3.73%	[3.21–4.33%]	0.6596
41–50	80	8.22%	4.68%	[3.78–5.79%]	50	62.50%	8.26%	[6.32–10.73%]	30	37.50%	2.72%	[1.91–3.86%]	0.0675
51–60	24	2.47%	3.88%	[2.62–5.71%]	12	50.00%	6.28%	[3.63–10.66%]	12	50.00%	2.81%	[1.61–4.85%]	0.7867
>60	7	0.72%	1.70%	[0.83–3.47%]	5	71.43%	2.62%	[1.12–5.98%]	2	28.57%	0.91%	[0.25–3.25%]	0.5386
**NG**	**340**	**100%**	**2.10%**	**[1.89–2.33%]**	**277**	**81.47%**	**4.51%**	**[4.02–5.06%]**	**63**	**18.53%**	**0.63%**	**[0.49–0.80%]**	**<0.0001**
≤ 20	32	9.41%	16.84%	[12.19–22.81%]	22	68.75%	26.19%	[17.98–36.48%]	10	31.25%	9.43%	[5.20–16.5%]	0.0517
21–30	147	43.24%	2.34%	[1.99–2.74%]	116	78.91%	4.67%	[3.91–5.57%]	31	21.09%	0.82%	[0.58–1.18%]	0.2892
31–40	114	33.53%	1.63%	[1.36–1.95%]	100	87.72%	3.87%	[3.19–4.68%]	14	12.28%	0.32%	[0.19–0.54%]	0.0352
41–50	29	8.53%	1.70%	[1.19–2.43%]	26	89.66%	4.30%	[2.95–6.23%]	3	10.34%	0.27%	[0.09–0.79%]	0.2356
51–60	15	4.41%	2.43%	[1.48–3.97%]	10	66.67%	5.24%	[2.87–9.37%]	5	33.33%	1.17%	[0.50–2.71%]	0.2422
>60	3	0.88%	0.73%	[0.25–2.12%]	3	100%	1.57%	[0.54–4.51%]	0	0%	0.00%	[0.00–1.72%]	1.0000

**Figure 1:**
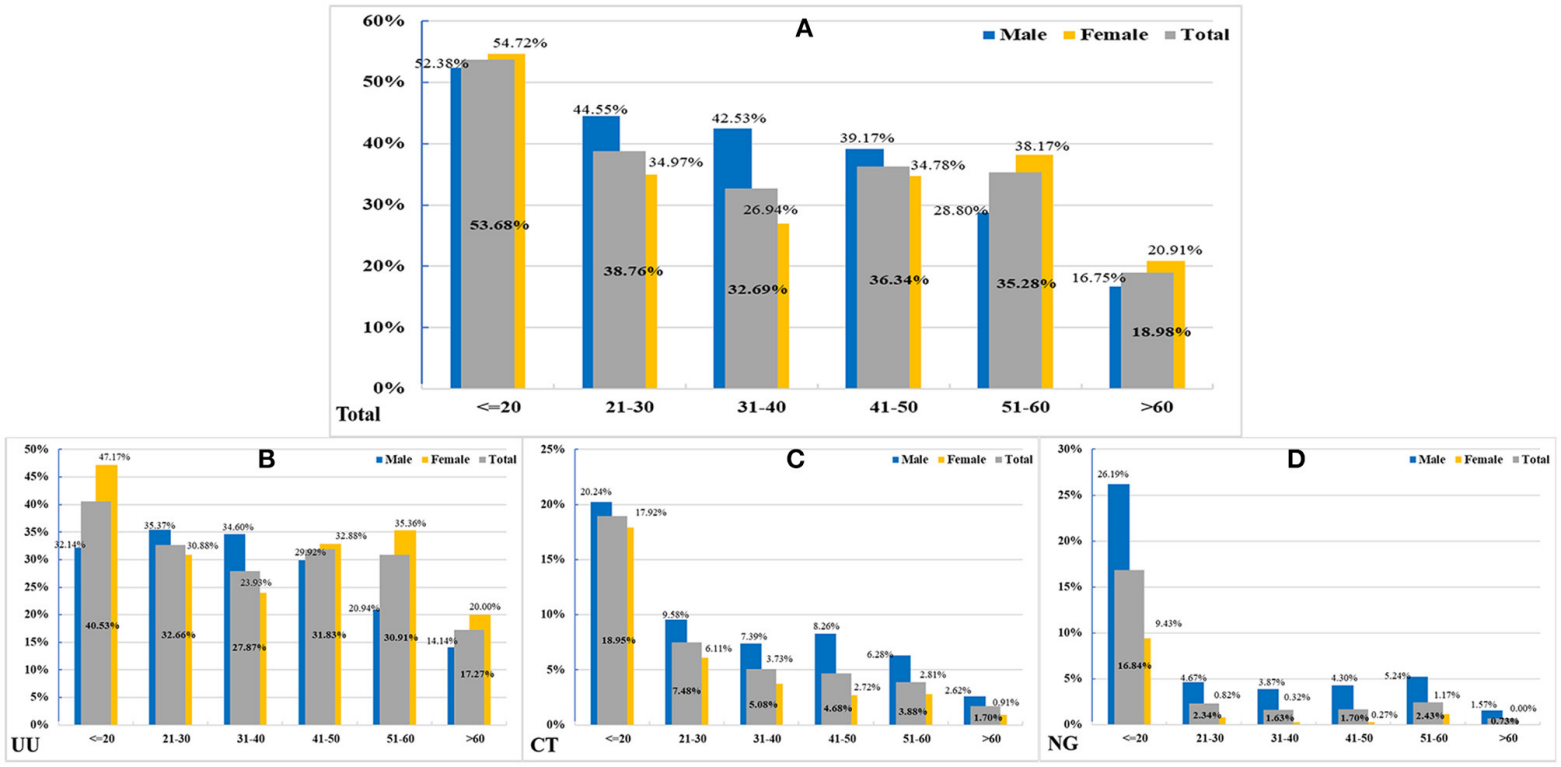
The positive detection rates (PDRs) in different age groups. **(A)** In 5,744 total positive patients in different genders (*Z* = 4.3292, *P* < 0.0001). **(B)** In 4,887 patients with UU infection (*Z* = 4.5677, *P* < 0.0001). **(C)** In 973 patients with CT infection (*Z* = 1.0633, *P* = 0.2877). **(D)** in 340 patients with NG infection (*Z* = 1.6276, *P* = 0.1036).

Patients were mostly 21–30 years old for all infections (UU: 41.99%; CT: 48.30%; NG: 43.24%). However, the highest PDRs of UU, CT, and NG infections were found in patients aged ≤ 20 years (40.53% [95% CI: 33.80–47.63%], 18.95% [14.01–25.12%], and 16.84% [12.19–22.81%]). The prevalence of UU infection significantly decreased with aging (*p* < 0.0001) (Figure 1).

**Figure 2:**
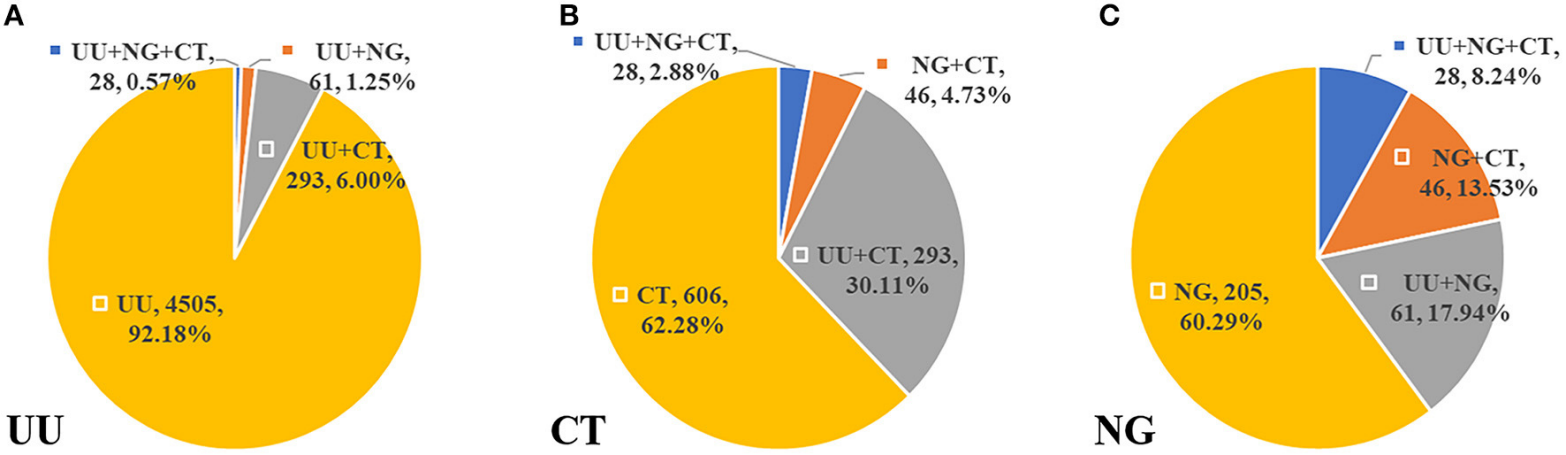
The distribution of co-infection patterns **(A)** in 4,887 UU infected cases. **(B)** In 973 CT infected cases. **(C)** In 340 NG infected cases.

Women constituted most (80.71%) of the 757 infected individuals who had no symptoms or complications (ages range: 7–78 years; 33.34 ± 9.47 years). A strong gender disparity was found in 41–50, 51–60, and >60 groups (*p* < 0.05) (Table 3). There were 428 (7.45%) cases of co-infection (ages range: 14–66 years, 29.98 ± 8.27 years), of which 57.01% were men. The most frequent co-infection pattern was UU+CT (68.46%), with patients aged 21–30 years having the highest incidence (56.31%) (Table 4). Among cases with CT or NG infection, the detection rates of UU were higher (32.99%, 26.18%) (Figure 2).

**Table 3:** The distribution of genders in 757 asymptomatic patients.

**Types**	**Total**		**Male**		**Female**		***p***
	***N***	**Proportion (%)**	***N***	**Proportion (%)**	***N***	**Proportion (%)**	
**Total**	**757**	100.00%	**146**	19.29%	**611**	80.71%	<0.0001
**Pathogens**	**799**	100.00%	**147**	18.40%	**652**	81.60%	<0.0001
UU	683	85.48%	133	90.48%	550	84.36%	0.0571
CT	94	11.76%	14	9.52%	80	12.27%	0.3506
NG	22	2.75%	0	0.00%	22	3.37%	0.0239
**Infection types**							
**Single**	715	94.45%	145	99.32%	570	93.29%	0.0043
UU	643	84.94%	132	90.41%	511	83.63%	0.0397
CT	58	7.66%	13	8.90%	45	7.36%	0.5299
NG	14	1.85%	0	0.00%	14	2.29%	0.0843
**Double**	39	5.15%	1	0.68%	38	6.22%	0.0066
UU+CT	34	4.49%	1	0.68%	33	5.40%	0.0134
UU+NG	3	0.40%	0	0.00%	3	0.49%	1.0000
CT+NG	2	0.26%	0	0.00%	2	0.33%	1.0000
**Triple**	3	0.40%	0	0.00%	3	0.49%	1.0000
**Age groups**							
≤ 20	16	2.11%	2	1.37%	14	2.29%	0.7495
21–30	321	42.40%	63	43.15%	258	42.23%	0.8390
31–40	293	38.71%	66	45.21%	227	37.15%	0.0727
41–50	78	10.30%	5	3.42%	73	11.95%	0.0023
51–60	33	4.36%	1	0.68%	32	5.24%	0.0155
>60	16	0.02114	9	6.16%	7	1.15%	0.0010

**Table 4:** The distribution of genders in mixed infections in different age groups.

**Infection types**	**Total**		**Male**		**Female**		***p***
	***N***	**Proportion (%)**	***N***	**Proportion (%)**	***N***	**Proportion (%)**	
**Total**	**428**	**100.00%**	**244**	**57.01%**	**184**	**42.99%**	**<0.0001**
**Double**	**400**	**93.46%**	**224**	**91.80%**	**176**	**95.65%**	**<0.0001**
**UU+CT**	**293**	**68.46%**	**144**	**59.02%**	**149**	**80.98%**	**<0.0001**
≤ 20	13	4.44%	6	46.15%	7	53.85%	0.8252
21–30	165	56.31%	77	46.67%	88	53.33%	0.3350
31–40	90	30.72%	50	55.56%	40	44.44%	0.1440
41–50	19	6.48%	9	47.37%	10	52.63%	0.8726
51–60	5	1.71%	1	20.00%	4	80.00%	0.3711
>60	1	0.34%	1	100.00%	0	0.00%	0.4915
**UU+NG**	**61**	**14.25%**	**43**	**17.62%**	**18**	**9.78%**	**0.0133**
≤ 20	6	9.84%	6	100.00%	0	0.00%	0.1665
21–30	36	59.02%	23	63.89%	13	36.11%	0.1748
31–40	9	14.75%	6	66.67%	3	33.33%	1.0000
41–50	7	11.48%	5	71.43%	2	28.57%	1.0000
51–60	3	4.92%	3	100.00%	0	0.00%	0.5484
>60	0	0.00%	-	-	-	-	-
**NG+CT**	**46**	**10.75%**	**37**	**15.16%**	**9**	**4.89%**	**0.0004**
≤ 20	8	17.39%	4	50.00%	4	50.00%	0.0359
21–30	21	45.65%	18	85.71%	3	14.29%	0.4777
31–40	13	28.26%	12	92.31%	1	7.69%	0.4100
41–50	2	4.35%	2	100.00%	0	0.00%	1.0000
51–60	2	4.35%	1	50.00%	1	50.00%	0.3565
>60	0	0.00%	-	-	-	-	-
**Triple (UU+NG+CT)**	**28**	**6.54%**	**20**	**8.2%**	**8**	**4.35%**	**0.0045**
≤ 20	8	28.57%	3	37.50%	5	62.50%	0.0223
21–30	6	21.43%	4	66.67%	2	33.33%	1.0000
31–40	10	35.71%	9	90.00%	1	10.00%	0.1937
41–50	2	7.14%	2	100.00%	0	0.00%	1.0000
51–60	1	3.57%	1	100.00%	0	0.00%	1.0000
>60	1	3.57%	1	100.00%	0	0.00%	1.0000

## 4 Discussion

The burden of STIs in China has increased in the past decades (1, 3, 16). STIs are transmitted predominantly via unprotected sexual contact and are closely associated with several urogenital diseases (12), but all STIs are preventable, and most can be effectively cured or treated in case of timely diagnosis (3). Therefore, early detection and surveillance are key cornerstones for controlling STIs. However, most studies only focused on the distribution of pathogens in women, especially in women from the Department of Obstetrics and Gynecology (17), and the long-term epidemiologic features of STI-related pathogens in Shanghai have been rarely reported (18, 19). Enrolling 16,216 outpatients from Shanghai, we described the prevalence of UU, CT, and NG infections from 2016 to 2021 and analyzed the infection types between different genders and age groups to facilitate the development of effective surveillance and prevention strategies for these infections.

Our results revealed the substantial increase in the number of tested women, which may also contribute to the difference in prevalences of STIs among men and women. Accordingly, our study suggested that the monitoring should also be strengthened in men. The prevalence of STIs in our study (35.42%) was higher than that among US adults (about 20% in 2018) (2), indicating that periodic STI surveillance is needed in Shanghai. Researchers have clarified that UU infection can cause inflammation of the female reproductive system (13). Perinatal UU and CT infections can cause abortion, premature delivery, and low birth weight (8, 13). UU and CT are frequent among infertile men and decrease the quality of semen (7, 20, 21). In this study, up to 25% of men with STIs exhibited infertility and adverse pregnancy outcomes, which were the most common complications. However, the latest Chinese guidelines did not recommend screening for STI-related pathogens among infertile couples (22). Hence, we suggest a screening program for infertile couples, especially for men.

Additionally, the overall prevalence rates of UU, CT, and NG in Shanghai were all higher than those from developed countries (2, 23–25), which may be attributed to effective control measures in these countries, such as popularization of sexual knowledge, attention to sexual hygiene, and regular screening of high-risk people. The proportion and prevalence of UU infection were both significantly higher than those of CT and NG infections, which was consistent with previous studies (13, 26, 27). In a study from Beijing, which included patients from 2013 to 2016, the prevalence of the three pathogens was similar to our findings, but in our study, UU and NG infections were more prevalent in men (28). These results indicate that the prevalence may vary in different regions, and the distribution of pathogens can be different in different populations.

Young age has been verified as a strong risk factor for STIs (29). Our results also showed that almost 80% of UU, CT, and NG infections occurred in young individuals aged 21–40 years, similar to those reported from Beijing and Zhejiang, China (28, 30). Nevertheless, we also found a higher prevalence in men than in women in the 21–40-year-old group, suggesting that men aged 21–40 years should undergo UU, CT, and NG screening. Notably, the UU infection was more prevalent among women aged 41–60 years, possibly due to the unique structure of genital tracts and endocrine factors among these women (28). In women aged more than 40 years, STI detection can be considered a supplement to routine physical examination.

In our study, a considerable proportion of infected people, especially women (nearly 20%), had no clinical symptoms, which is consistent with previous findings claiming that female STI is more likely to be asymptomatic (3, 31). Notably, the mean age of co-infected patients (29.98 years) was lower than that of all infected patients (33.23 years), and men were significantly more susceptible to mixed infection, which may be related to the openness to sexuality and the increasing number of young MSM (YMSM) (32, 33). These might reflect a lack of sexual protection and the necessity of sex education, especially among YMSM. Some studies have reported the relationships between STI pathogens. For instance, STI pathogens can increase the risk of HIV acquisition and transmission (28); UU is associated with the persistence of HPV infection and early cytological changes of the cervix (34); and CT colonization contributes to UU or NG infection (30). In our study population, the UU+CT co-infection pattern was dominant, especially among women, where UU played a major role in co-infections. In our study, the distribution of co-infection patterns in women was similar to that in Zhejiang (30). The high prevalence in patients aged 21–30 years suggests that surveillance should be strengthened in this population, and people in their 40s should be cautious of multiple STIs.

In general, UU accounted for the highest proportion of STIs, but its prevalence decreased with aging. The prevalence and infection types varied in age and gender groups. People in their 20s and 30s were most infected with UU, CT, and NG, and the three pathogens were all more prevalent in men than in women. Our findings suggest the screening of UU, CT, and NG for high-risk populations, such as young men, asymptomatic women over 40 years old, and infertile couples. In addition, we illuminated the relationship between gender and STIs to promote the early diagnosis and treatment of STIs and avoid adverse outcomes.

To the best of our knowledge, this was the first study investigating the prevalence of UU, CT, and NG infections among outpatients in Shanghai and analyzing their distribution in different gender and age groups. Information on education, STI history, sexual orientation, condom usage, and sexual partners was not collected, which is the main limitation of this study. The prevalence reported by this study might not be comprehensive enough; thus, further studies are needed to assess the risk factors for these STI-related pathogens in Shanghai and other regions in China.

## 5 Conclusion

We demonstrated the high prevalence of STIs in Shanghai and the predominant role of UU. Young men aged ≤ 20 years showed the highest STI prevalence. Different screening, prevention, and treatment strategies are needed for different gender and age groups.

## Data availability statement

The original contributions presented in the study are included in the article/Supplementary material, further inquiries can be directed to the corresponding authors.

## Ethics statement

This study was approved by the Ethics Committee of Huadong Hospital Affiliated to Fudan University (Ethics Approval Number: 20190112). The studies were conducted in accordance with the local legislation and institutional requirements. The participants provided their written informed consent to participate in this study.

## Author contributions

SuW, HZ, and ShW contributed to conception and design of the study. SuW and JM organized the database. SuW, LD, YL, ZS, WJ, and YM performed the statistical analysis. SuW wrote the first draft of the manuscript. HZ, JM, and ShW wrote sections of the manuscript. All authors contributed to manuscript revision, read, and approved the submitted version.
