# Supplementary material for: Characteristics of common pathogens of urogenital tract among outpatients in Shanghai, China from 2016 to 2021
Source: Front Public Health. 2023 Nov 27;11:1228048. doi: 10.3389/fpubh.2023.1228048 (PMC10711282; doi:10.3389/fpubh.2023.1228048)
Supplement: Supplementary file 1 [file Table_1.docx]

Supplementary Material

Epidemiological Characteristics of Common Pathogens in Urogenital Tract in the Outpatients from Shanghai, 2016-2021

Su Wang^1,2,3†^, Li Ding^1,2,3†^, Yixin Liu^4†^, Zhaoyang Sun^1,2,3†^, Yingying Sun^1^, Wenrong Jiang^1,2,3^, Yingxin Miao^1,2,3^, Shiwen Wang^1,2,3*^, Jun Meng^5*^, Hu Zhao^1,2,3*^

*** Correspondence:** Hu Zhao, [hubertzhao@163.com](mailto:hubertzhao@163.com); Jun Meng, [mj40563@rjh.com.cn](mailto:mj40563@rjh.com.cn); Shiwen Wang, wangshiwen1986@aliyun.comedu.

# Supplementary Tables

## Supplementary Table

**Supplementary Table S1.** **The general characteristics of genders in 16216 enrolled patients.**

| **Group** | **Total** | | **Male** | | **Female** | | ***p*** |
| --- | --- | --- | --- | --- | --- | --- | --- |
|  | **N** | **%** | **N** | **%** | **N** | **%** |  |
| **Total** | **16216** | **100%** | **6140** | **37.86%** | **10076** | **62.14%** | **<.0001** |
| **Year** |  |  |  |  |  |  |  |
| 2016 | 976 | 6.02% | 764 | 78.28% | 212 | 21.72% | <.0001 |
| 2017 | 1574 | 9.71% | 1085 | 68.93% | 489 | 31.07% | <.0001 |
| 2018 | 2568 | 15.84% | 1063 | 41.39% | 1505 | 58.61% | <.0001 |
| 2019 | 2557 | 15.77% | 1120 | 43.80% | 1437 | 56.20% | <.0001 |
| 2020 | 3094 | 19.08% | 907 | 29.31% | 2187 | 70.69% | <.0001 |
| 2021 | 5447 | 33.59% | 1201 | 22.05% | 4246 | 77.95% | <.0001 |
| **Age** |  |  |  |  |  |  |  |
| <=20 | 190 | 1.17% | 84 | 44.21% | 106 | 55.79% | 0.0696 |
| 21-30 | 6283 | 38.75% | 2485 | 39.55% | 3798 | 60.45% | 0.0004 |
| 31-40 | 7005 | 43.20% | 2584 | 36.89% | 4421 | 63.11% | 0.0255 |
| 41-50 | 1709 | 10.54% | 605 | 35.40% | 1104 | 64.60% | 0.0265 |
| 51-60 | 618 | 3.81% | 191 | 30.91% | 427 | 69.09% | 0.0003 |
| >60 | 411 | 2.53% | 191 | 46.47% | 220 | 53.53% | 0.0003 |
| **Department** |  |  |  |  |  |  |  |
| Gynaecology | 7370 | 45.45% | - | - | 7370 | 100% | - |
| Urology | 3985 | 24.57% | 3518 | 88.28% | 467 | 11.72% | <.0001 |
| Reproductive center | 3715 | 22.91% | 1641 | 44.17% | 2074 | 55.83% | <.0001 |
| Andrology | 684 | 4.22% | 684 | 100.00% | - | - | - |
| Physical examination center | 320 | 1.97% | 272 | 85.00% | 48 | 15.00% | <.0001 |
| Pain | 99 | 0.61% | 1 | 1.01% | 98 | 98.99% | <.0001 |
| Others | 43 | 0.27% | 24 | 55.81% | 19 | 44.19% | 0.0151 |
